# Blood monocyte levels predict the risk of acute exacerbations of chronic obstructive pulmonary disease: a retrospective case–control study

**DOI:** 10.1038/s41598-022-25520-8

**Published:** 2022-12-06

**Authors:** Ching-Hsiung Lin, Yi-Rong Li, Pei Ru Lin, Bing-Yen Wang, Sheng-Hao Lin, Kuo-Yang Huang, Chew-Teng Kor

**Affiliations:** 1 Department of Internal Medicine, Division of Chest Medicine, Changhua Christian Hospital, 135 Nanhsiao Street, Changhua, 50006 Taiwan; 2 Institute of Genomics and Bioinformatics, National Chung Hsing University, Taichung, Taiwan; 3 Ph.D. Program in Translational Medicine, National Chung Hsing University, Taichung, Taiwan; 4 Department of Recreation and Holistic Wellness, MingDao University, Changhua, Taiwan; 5 Thoracic Medicine Research Center, Changhua Christian Hospital, Changhua, 500 Taiwan; 6 Big Data Center, Changhua Christian Hospital, Changhua, 500 Taiwan; 7 Department of Surgery, Division of Thoracic Surgery, Changhua Christian Hospital, Changhua, 500 Taiwan; 8 Graduate Institute of Statistics and Information Science, National Changhua University of Education, Changhua, 500 Taiwan

**Keywords:** Immunology, Biomarkers, Medical research, Risk factors, Signs and symptoms

## Abstract

Monocytes were critical cells in the innate immune system. Monocyte recruitment to the lungs is a crucial process of pathophysiology in chronic obstructive pulmonary disease (COPD). Current evidence on the association between the occurrence of acute exacerbations of COPD (AECOPD) and monocytes was unclear. This study aimed to examine whether blood monocytes are associated with the occurrence of AECOPD and to determine the specific blood monocyte level to predict AECOPD. A retrospective case–control study was conducted at Changhua Christian Hospital. A total of 444 eligible patients with COPD were included between January 2017 and December 2019. Restricted cubic splines were used to analyze the nonlinear relationships between continuous white blood cell values and the occurrence of AECOPD. The association between monocytes and the occurrence of AECOPD was assessed using the logistic, lasso, and ridge regression models. Restricted cubic splines revealed nonlinear associations among the monocyte level, the continuous value of the eosinophil-to-lymphocyte ratio, and the occurrence of AECOPD. The lowest risk of occurrence of AECOPD ranged from 7.4 to 10%; < 7.4% with an absolute count < 0.62 or > 10% indicated significant risk. No significant association was noted between the eosinophil-to-lymphocyte ratio categories in the tertiles (< 0.049, 0.049 to < 0.122, and ≥ 0.122) and the risk of AECOPD. A significantly higher risk was noted in the association of the occurrence of AECOPD with the CAT score; mMRC score; wheezing cough; preexisting chronic pulmonary disease; hypertension and malignancy; use of dual- and triple, and oral long-acting bronchodilators for COPD treatment; and WBC count. We reported a nonlinear relationship between monocytes and the occurrence of AECOPD. Patients with monocyte percentage of > 10% or < 7.4% with an absolute count < 0.62 had higher risk of occurrence of AECOPD. Overall, our study demonstrated the specific value of monocytes in identifying high risks of the occurrence of AECOPD; this value is an easy-to-obtain, inexpensive biomarker in patients with AECOPD and should be further investigated in future prospective clinical studies.

## Introduction

Acute exacerbations of chronic obstructive pulmonary disease (AECOPD) were responsible for worsening lung function, poor quality of life, increased use of emergency health care, and COPD-related mortality^1–3^. AECOPD were heterogeneous in terms of airway inflammation and etiology^4–6^. Recent studies had focused on the role of the counts of blood granulocytes, including neutrophils and eosinophils, as predictors of AECOPD^7–9^. Besides granulocytes, monocytes are key innate immune system cells, and their recruitment to the lungs is an important step in COPD pathogenesis^10–13^. The number of circulating monocytes, specifically that of nonclassical monocytes, was considerably elevated in patients with severe COPD^14^. However, evidence regarding the association between blood monocytes and AECOPD is scarce.

A previous study that performed linear regression analysis reported that the total monocyte percentage or the monocyte count was not positively correlated with the length of hospital stay among patients with AECOPD^15^. By contrast, evidence indicated the importance of blood monocyte count in airway disease. A previous study reported that elevated monocyte counts are associated with increased rates of idiopathic pulmonary fibrosis^16^. Moreover, our previous study found that monocyte counts were positively correlated with the occurrence of AECOPD and were designated as a key feature for AECOPD prediction through SHAPley Additive exPlanations (SHAP) in a gradient-boosting machine-based machine learning model^17^. Contrary to linear regression plots, SHAP value plots reveal the important nonlinear relationships between independent and dependent variables^18^. Therefore, based on our previous findings, we hypothesized that there exists a nonlinear relationship between monocyte levels and AECOPD.

This study examined whether blood monocyte levels are associated with the occurrence of AECOPD and their use as a potential biomarker for helping to predict AECOPD. We first evaluated whether a nonlinear relationship exists between monocyte levels and the occurrence of AECOPD and then examined the special value of blood monocytes to predict the occurrence of AECOPD.

## Methods

### Study participants

**Figure 1:**
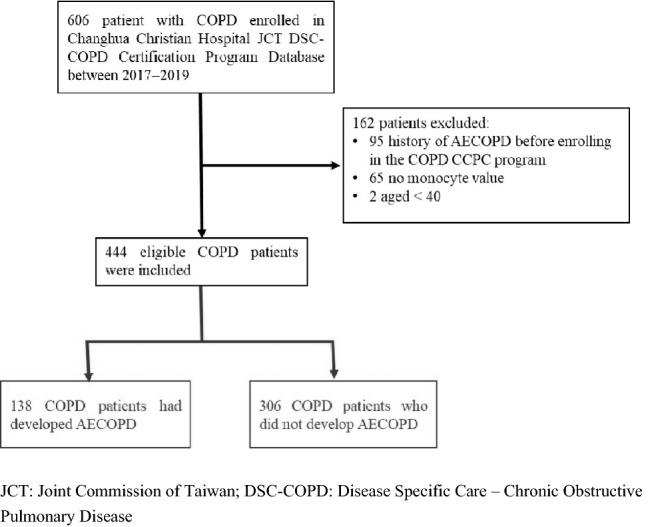
Flowchart of the study protocol.

This retrospective case–control study was conducted at Changhua Christian Hospital (CCH), Changhua, Taiwan. A total of 606 patients with COPD were recruited between January 2017 and December 2019 based on data from the CCH Clinical Research Database, an integrated database of all electronic medical record systems, including the Joint Commission of Taiwan: Disease Specific Care–Chronic Obstructive Pulmonary Disease Certification Database; inpatient care records; prescriptions; laboratory data; clinical visit records; and death records. All patients were diagnosed using the *International Classification of Diseases, 10th edition* (*ICD10*) diagnosis codes (J41–J44). The diagnosis in outpatients was confirmed using a spirometer (postbronchodilator FEV1/FVC < 70%) within 90 days. Patients met the eligibility criteria if they listed the *ICD-10* diagnostic codes J41–J44 after receiving outpatient treatment from CCH. The criteria were confirmed using spirometry within 90 days before diagnosis. Patients with a history of AECOPD before enrolling in the JCT DSC-COPD certification program (n = 95) in previous 1 year, those aged < 40 years (n = 2), and those for whom monocyte values were not available (n = 65) were excluded (Fig. 1). We assessed history of AECOPD by reviewing patients’ clinical outpatient record who had prescribed systemic steroids with or without antibiotics and inpatient care record who had emergency room visits or admission due to COPD. Finally, 444 eligible patients were included in the study. The study timeline of this study is shown in Supplemental Figure S1. In brief, the AECOPD event was the earliest event from the date of monocyte count collection. The period of study was 1 years after program enrollment. The study was conducted according to the guidelines of the Declaration of Helsinki, and the Institutional Review Board of Changhua Christian Hospital waived off the requirement for informed consent and approved the study (IRB No: 220608). All patient records and data were deidentified and anonymized prior to analysis.

### Main exposure and other confounders

The monocyte trisection value for our patient with COPD was 7.5–10%. The patients were classified into low (< 7.5%), medium (7.5 to < 10%), and high (≥ 10%) monocyte groups based on established cutoff values. For each patient, the baseline data at enrollment included demographic data (sex, age, body mass index, and smoking habits) and clinical characteristics (post-bronchodilator spirometry test, CAT scores, mMRC dyspnea scores, and respiratory symptoms). Comorbidities included diabetes mellitus, hypertension, hyperlipidemia, congestive heart failure, sleep disorder, asthma, cancer, pneumonia, and chronic lung disease. Types of chronic lung disease included pulmonary fibrosis, pneumonitis, asthma and other lung conditions. The analyzed laboratory parameters included blood levels of eosinophils, lymphocytes, and neutrophils as well as eosinophil-to-lymphocyte ratio (ELR), neutrophil-to-lymphocyte ratio (NLR), platelet count, WBC count, RBC count, hemoglobin level, red cell distribution width, pH, basophil level, and mean platelet volume. Blood samples were collected and analyzed by Beckman Coulter DxH 900 hematology analyzer (Beckman Coulter, Miami, FL). The use of COPD treatments (mono-, dual-, and triple-therapy; oral long-acting bronchodilators; and methylxanthines) was also assessed. Potential confounders were included from the CCH Clinical Research Database.

### Outcomes

The occurrence of AECOPD was defined as an outpatient visit, emergency room visit, or admission with an *ICD-10* code of COPD (J43.x-44.x, except J430, within a fifth secondary diagnosis in outpatient setting or as emergency room visits, or as the primary diagnosis after hospital admission) at which time systemic steroids (ATC code: H02) with or without antibiotics (ATC code: J01) were prescribed.

### Statistical analysis and logistic regression regularization

Categorical variables are expressed as a percentage and continuous variables are expressed as medians and interquartile ranges (IQRs). The chi-square test was used to compare categorical variables, and the Mann–Whitney U test was used to compare continuous variables. Restricted cubic splines (RCSs) served as a flexible tool for modeling and presenting complex, nonlinear relationships between continuous values of WBC types and AECOPD. The P values in nonlinear regression were calculated using likelihood ratio tests that compared the model with only a linear term against the model with linear and cubic spline terms.

Boxplot analysis was used as a data visualization method to present (1) the median monocyte values for the AECOPD and non-AECOPD groups and (2) the stratification according to low, medium, and high monocyte groups. The prevalence of AECOPD stratified by monocyte categories is also presented using a bar graph. The association between the monocyte group and the occurrence of AECOPD was assessed using logistic regression. The odds ratio and 95% confidence interval (95% CI) were calculated using crude and multivariate analyses. The variables used in the multivariate logistic regression were selected based on the crude analysis (p < 0.05). To improve the robustness of our results, the lasso and ridge regression models were used to select the best variables, and the regularization parameter was set as the minimum lambda to minimize the tenfold cross-validation prediction error rate, which predicts the most accurate model. All data were analyzed using SAS, and a visualization plot was generated using R software (version 4.1.0; The Comprehensive R Archive Network: http://cran.r-project.org). Two-sided P values of < 0.05 were considered statistically significant.

### Ethical approval and consent to participate

The Institutional Review Board of CCH waived off the requirement for informed consent and approved the study (IRB No: 220608). All patient records and data were deidentified and anonymized prior to analysis.

## Results

### Study characteristics

A total of 444 patients with COPD were included in this study. We reviewed the electronic medical records (EMR) of these patients and observed that 138 (31.1%) of the 444 had developed AECOPD during the program and 306 (68.9%) COPD patients who did not develop AECOPD, and their median monocyte percentage was 8.70 [IQR 6.60–10.60]. The median patient age was 73 years. Of the 444 patients, 394 (88.7%) were men; their median CAT score was 4 (3, 6) as well as 60 (51, 67) FEV1/FVC (%) results after the bronchodilator test. Furthermore, 32.9% (n = 146), 33.1% (n = 147), and 34.0% (n = 151) of the patients had monocyte percentage of < 7.5%, 7.5 to < 10%, and ≥ 10%, respectively.

Table 1 listed the baseline characteristics of the patients stratified by the occurrence of AECOPD. Patients with AECOPD were exposed to groups of low and high monocyte percentage, but no significant differences were found in the continuous value of monocyte percentage between the two groups. However, patients with AECOPD had significantly higher levels of monocyte count and WBC count, but the levels of eosinophil, lymphocyte, neutrophil, neutrophil-to-lymphocyte, and eosinophil-to-lymphocyte levels were not significantly different. Other laboratory data also did not show significant differences between the two groups (Table 1).

**Table 1:** Demographic characteristics of the study population.

	No AECOPD	AECOPD	P-value
Sample size	306	138	
Monocyte%	8.6(6.7,10.4)	8.7(6.5,11.2)	0.605
Low at < 7.5%	95(31.05%)	51(36.96%)	0.003
Medium at 7.5 to < 10%	117(38.24%)	30(21.74%)	
High at ≥ 10%	94(30.72%)	57(41.3%)	
Monocyte count	60.62(44.16,79.92)	67.08(51.12,94.01)	0.004
FEV1_post	1.43(1.06,1.85)	1.23(0.87,1.63)	0.001
FEV1_post%	61.95(47.9,75.5)	53.9(39.5,66.8)	< 0.001
FEV1/FVC_post%	61.13(52.66,67.12)	59.53(50.28,65.27)	0.035
CAT	3(2,5)	5(4,8)	< 0.001
≤ 10	303(99.02%)	128(92.75%)	< 0.001
> 10	3(0.98%)	10(7.25%)	
MMRC	2(1,2)	2(1,2)	0.110
0	36(11.76%)	5(3.62%)	0.055
1	100(32.68%)	48(34.78%)	
2	144(47.06%)	73(52.9%)	
≥ 3	26(8.5%)	12(8.7%)	
Gender, Male	270(88.24%)	124(89.86%)	0.617
Age	72(65,81)	74(67,81)	0.383
Cough	150(49.02%)	100(72.46%)	< 0.001
Dyspnea	116(37.91%)	78(56.52%)	< 0.001
Wheeze	95(31.05%)	84(60.87%)	< 0.001
Chest tight	43(14.05%)	21(15.22%)	0.746
Chest pain	24(7.84%)	14(10.14%)	0.422
HPT	144(47.06%)	82(59.42%)	0.047
DM	68(22.22%)	39(28.26%)	0.317
Hyperlipidemia	74(24.18%)	33(23.91%)	0.795
CHF	15(4.9%)	17(12.32%)	0.016
CPD	157(51.31%)	96(69.57%)	< 0.001
Heart failure	50(16.34%)	27(19.57%)	0.572
Sleep disorder	99(32.35%)	61(44.2%)	0.016
malignancy	44(14.38%)	30(21.74%)	0.128
Pneumonia	34(11.11%)	37(26.81%)	< 0.001
Eosinophil	1.7(0.8,3.4)	1.45(0.5,3.5)	0.495
Lymphocyte	21.3(13.4,30)	19.8(11,28.5)	0.106
Neutrophil	65(56.3,76.1)	67.3(55.8,79.3)	0.353
Eosinophil to Lymphocyte	0.08(0.04,0.15)	0.08(0.03,0.18)	0.927
< 0.049	110(35.95%)	40(28.99%)	0.357
0.049 to < 0.122	98(32.03%)	49(35.51%)	
> = 0.122	98(32.03%)	49(35.51%)	
Neutrophil to Lymphocyte	3(1.88,5.63)	3.34(1.98,7.21)	0.146
Platelet count	205.5(162,246)	195.5(161,237)	0.368
WBC count	7(5.6,9.1)	7.95(6.4,10.3)	< 0.001
RBC count	4.51(4.09,4.86)	4.55(4.22,4.86)	0.260
Hb	13.75(12.2,15)	13.9(12.8,15)	0.358
RDW	13.85(13.3,14.5)	13.9(13.4,14.7)	0.540
pH	6.68(6,7.32)	7(6,7.38)	0.428
Basophil	0.5(0.4,0.8)	0.6(0.4,0.8)	0.849
MPV	8(7.4,8.6)	8.1(7.5,8.9)	0.306
CRP	0.77(0.13,3.85)	0.62(0.14,4.21)	0.828
COPD medication within 6 months			
Mono- therapy	46(15.03%)	25(18.12%)	0.412
Dual- therapy	191(62.42%)	95(68.84%)	0.191
Triple- therapy	51(16.67%)	46(33.33%)	< 0.001
Oral long-acting bronchodilators	14(4.58%)	21(15.22%)	< 0.001
Methylxanthines	182(59.48%)	108(78.26%)	< 0.001
SBP	133.8(125,145.4)	133(124,145)	0.973
DBP	75(68,81)	76(70,82)	0.289
Pulse rate	83(75,92)	84(74,95)	0.650
Breathing	18(18,20)	18(18,19)	1.000
BMI	23.82(21.21,26.17)	23.38(21.19,26.01)	0.490
Smoking status			
Never smoker	66(21.57%)	27(19.57%)	0.605
Former smoker	194(63.4%)	94(68.12%)	
Current smoker	46(15.03%)	17(12.32%)	

### Nonlinear relationship between WBC types and AECOPD

**Figure 2:**
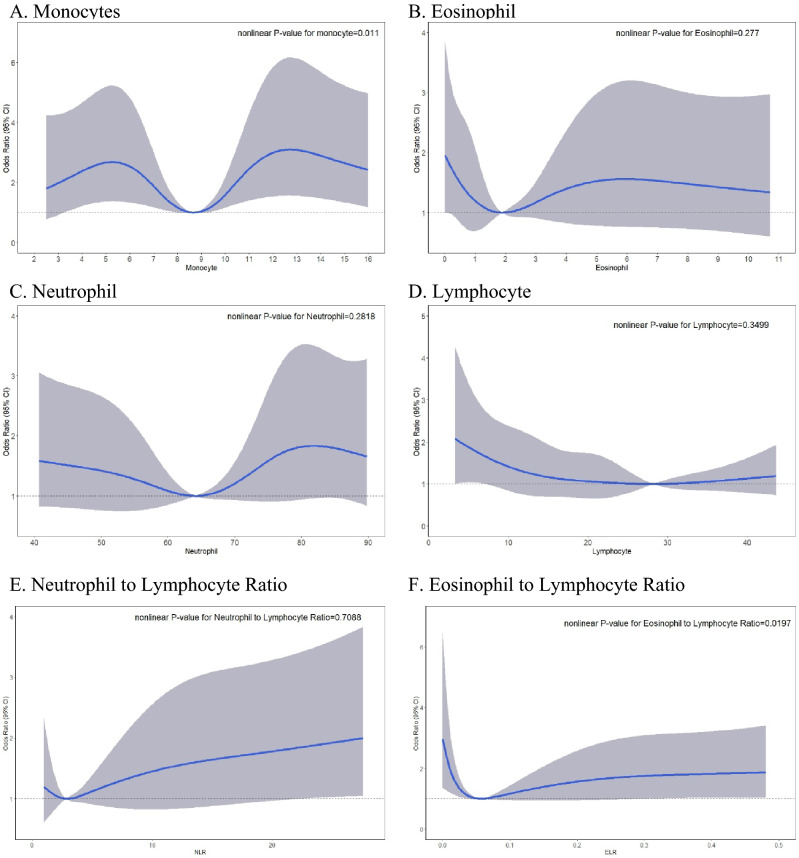

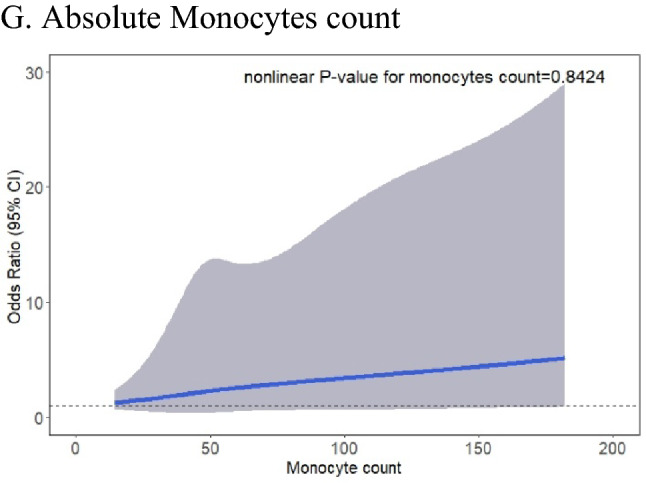
Restricted cubic spline graph showing the association between white blood cell types and the occurrence of AECOPD in patients with COPD (AECOPD, n = 138; non-AECOPD, n = 306). Gray shaded areas indicate 95% CIs.

The RCS graph demonstrated a nonlinear association between continuous values of WBC types and the occurrence of AECOPD in patients COPD (Fig. 2). After observing the possibility of a non-linear relationship, further logistic regression models were performed to assess the effect of WBC type categories on AECOPD risk. RCSs revealed nonlinear associations between the monocyte percentage level, the continuous value of ELR, and the occurrence of AECOPD (P nonlinearity = 0.011 for monocytes percentage level and 0.019 for the continuous value of ELR), and the results yielded a U-shaped graph (Fig. 2). The lowest risk of the occurrence of AECOPD was between 7.4% and 10%; values < 7.4% and > 10% indicated significant risk. Although the continuous value of ELR results also yielded a U-shaped graph, the association between ELR categories in the tertiles and the occurrence of AECOPD was not significant (P > 0.05; Supplemental Table S1). We also analyzed the relationship between individual absolute monocyte counts and AECOPD and found no nonlinearity (P = 0.842 in Fig. 2).

### Special value of blood monocytes for predicting AECOPD

**Figure 3:**
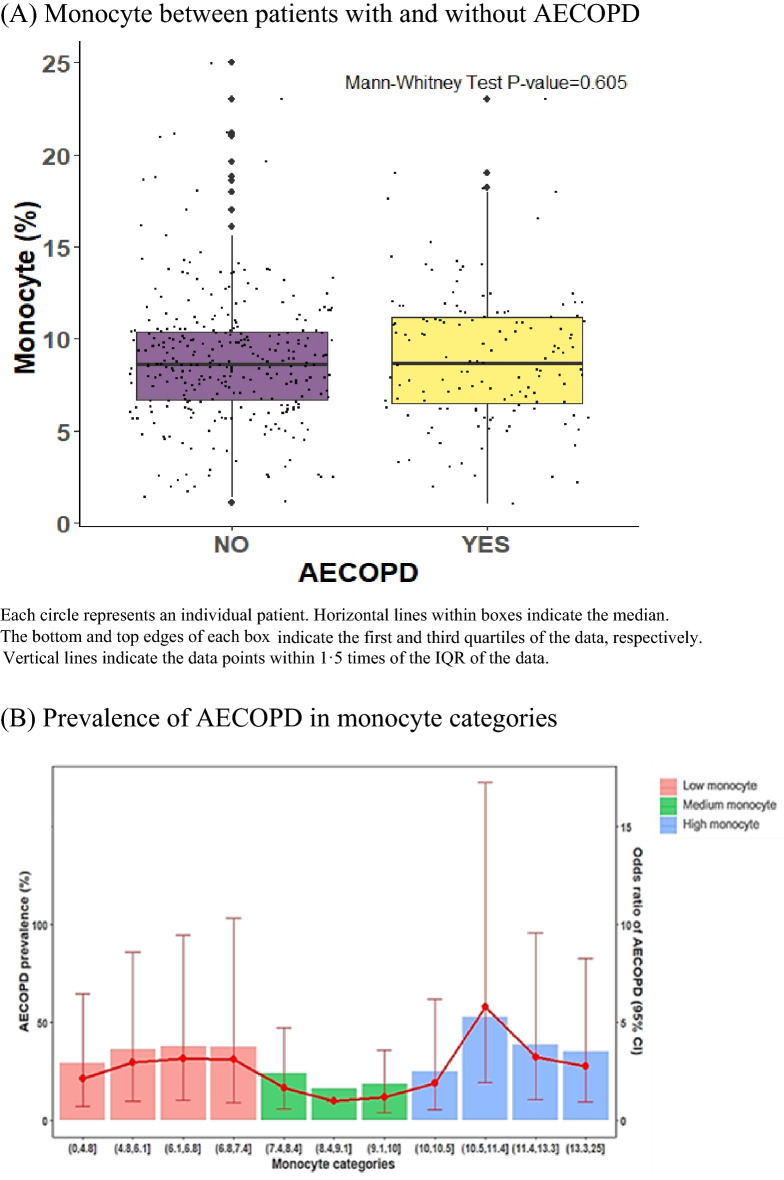

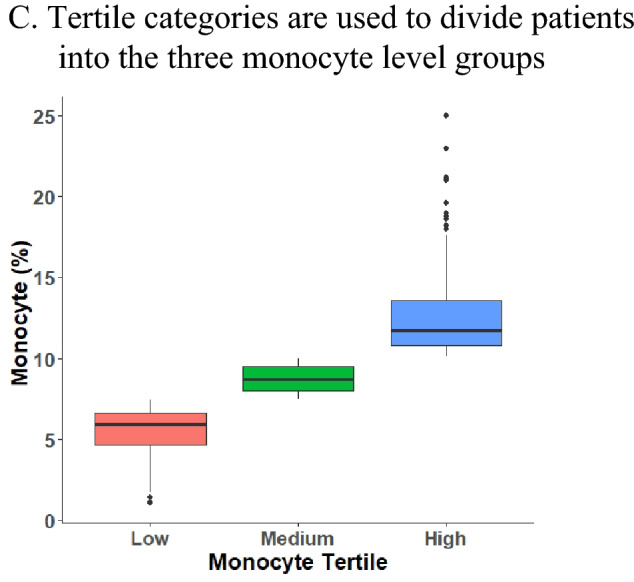
Association of monocyte with AECOPD and grouping (AECOPD, n = 138; non-AECOPD, n = 306).

The box plot (Fig. 3A) revealed no significant differences in the median monocyte percentage between AECOPD and non-AECOPD patients. A nonlinear relationship rendered the monocyte effect intangible as demonstrated in two-sample comparisons. The lowest risk of the occurrence of AECOPD was noted in patients with COPD having monocyte percentage between 7.4% and 10%; below and above this range, the risk of the occurrence of AECOPD gradually kept increasing (Fig. 3B). The corresponding median and IQRs for the low, medium, and high monocyte groups were 5.9 (4.6–6.6), 8.6 (8.0–9.4), and 11.5 (10.6–13.6), respectively (Fig. 3C). In the unadjusted model, a U-shaped curve was noted between monocytes percentage and the occurrence of AECOPD (low group: OR 2.09; 95% CI 1.24–3.54; P = 0.006 and high group: OR 2.37; 95% CI 1.41–3.97; P = 0.001). However, using the adjusted model, we were unsure if the association was significant in the low monocyte group (OR 1.47; 95% CI 0.76–2.86; P = 0.256) but it was certainly significant in the high monocyte group (OR 2.70; 95% CI 1.42–5.13; P = 0.002). The results of the lasso and ridge regression regularizations were consistent with those of the multivariate-adjusted analysis (Supplemental Table S2). Moreover, we also analyzed the relationship between individual absolute monocyte counts and AECOPD, and we found that this relationship is positively correlated rather than nonlinear. In both unadjusted and adjusted model, the risk of AECOPD increased when the absolute monocyte counts increased (OR 2.03; 95% CI 1.23–3.35; P = 0.006 for unadjusted model; OR 2.09; 95% CI 1.16–3.78; P = 0.014 for adjusted model, Table 2). Similarly, the risk of AECOPD was also increased in groups with higher absolute monocyte counts (Table 2).

**Table 2:** Crude analysis and adjusted odds ratio for the occurrence of AECOPD according to monocyte percentage.

	Unadjusted Model		Adjusted Logistic Model	
	cOR (95% CI)	P-value	aOR (95% CI)	P-value
Monocyte percentage in trisection				
Low	2.09 (1.24,3.54)	0.006	1.47 (0.76,2.86)	0.256
Medium	Reference		Reference	
High	2.37 (1.41, 3.97)	0.001	2.70 (1.42,5.13)	0.002
Absolute Monocyte Count				
Absolute Monocyte count	2.03 (1.23,3.35)	0.006	2.09 (1.16,3.78)	0.014
Absolute Monocyte count in the median				
Low (< 0.62)	1		1	
High (≥ 0.62)	1.46 (0.98,2.19)	0.066	1.79 (1.08,2.97)	0.024
Absolute Monocyte count in the tertile				
Low (< 0.52)	1		1	
Medium (0.52 to < 0.76)	1.32 (0.79,2.20)	0.296	2.24 (1.16,4.31)	0.016
High (≥ 0.76)	2.01 (1.22,3.31)	0.006	2.80(1.48,5.29)	0.001

Model adjusted for FEV1, CAT, MMRC, symptom (cough, dyspnea, wheeze), medication use (oral LABA, methylxanthines), comorbidities (HPT, CHF, CPD, sleep disorder, malignancy, Pneumonia), lab data (WBC count, NLR), COPD prescription (COPD triple), these variables were selected from the crude model with p-value < 0.05.

All models have performed the multivariate logistic regression model with a backward eliminating process.

### AECOPD risk matrix of absolute monocyte count and monocytes percentage

**Figure 4:**
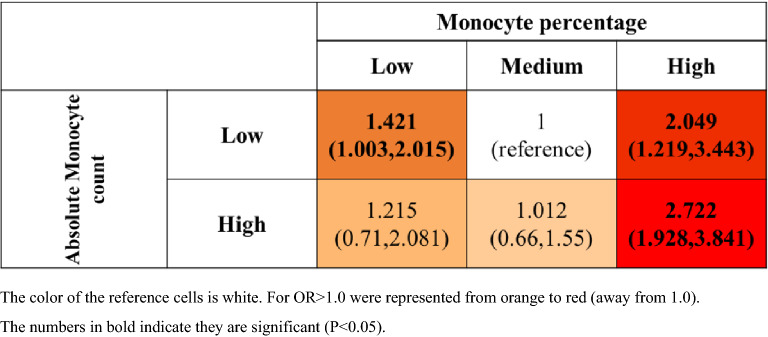
Risk matrices showing the adjusted ORs for AECOPD by using monocyte percentage categories and absolute monocyte count in the median (AECOPD, n = 138; non-AECOPD, n = 306).

Figure 4 presented the AECOPD risk matrix determined by using monocyte percentage categories and absolute monocyte count in the median. The results gave us insight into the increased risk of AECOPD when both the absolute monocyte count and the percentage of monocytes were low. Additionally, the risk of AECOPD increased in the group with a high percentage of monocytes, regardless of absolute monocyte count. However, no increased risk of AECOPD was found in the high-absolute-monocyte-count group when the monocyte percentage was low and medium.

### Other significant risk factors for AECOPD occurrence

**Figure 5:**
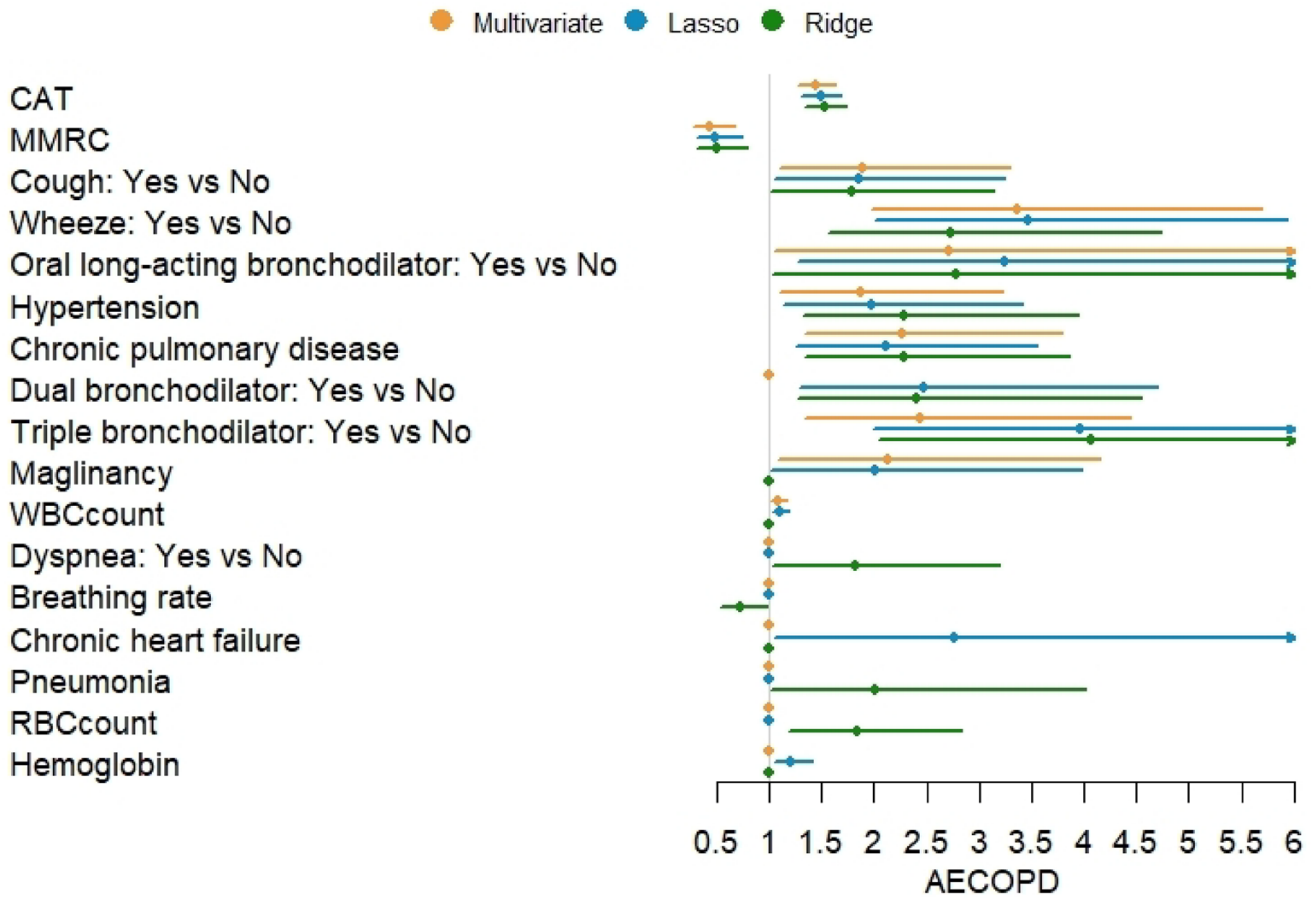
Other significant risk factors for the occurrence of AECOPD. (AECOPD, n = 138; non-AECOPD, n = 306).

Figure 5 showed the associations between other factors and the occurrence of AECOPD. A significantly higher risk was noted in the association of the occurrence of AECOPD with the CAT score; mMRC score; wheezing and cough; preexisting chronic pulmonary disease; hypertension and malignancy; use of dual- and triple-therapy bronchodilators and oral long-acting bronchodilators; and WBC count. According to lasso regression, chronic heart disease and hemoglobin levels were significantly positively associated with the occurrence of AECOPD. In the ridge regression model, dyspnea symptoms, pneumonia, and RBC count were significantly positively correlated with the occurrence of AECOPD, whereas respiratory rate was significantly negatively correlated with the occurrence of AECOPD.

## Discussion

This retrospective study revealed that both low (< 7.5%) and high (≥ 10%) monocyte percentage were associated with an increased risk of the occurrence of AECOPD. A U-shaped association was noted between the occurrence of AECOPD and the monocyte percentage but not the levels of eosinophils, neutrophils, and lymphocytes or NLR. The relationship between individual absolute monocyte count and the AECOPD was found to be linear rather than nonlinear. However, focusing only on the linear relationship between monocyte count and the AECOPD was insufficient to assess the critical role of monocytes, as more attention is paid to monocytosis and the effect of monocytopenia on AECOPD was easily overlooked. White blood cells contain five types of leukocyte cells (neutrophils, lymphocytes, monocytes, eosinophils, and Basophils). Absolute monocyte count was highly correlated with WBC count (spearmen rho correlation = 0.622, p-value < 0.001 in our study). Monocyte percentage was used to measure the number of monocytes in percentage to other types, and it could be adequately used to measure whether there were more or less than other types of white blood cells. To more accurately assess the overall impact of monocytes on the risk of AECOPD, risk matrix analysis provided insight into the significant nonlinear effect of monocytes on the risk of AECOPD. To the best of our knowledge, this study is the first to examine the nonlinear relationship between monocyte level and the occurrence of AECOPD. Our study provided evidence that the association between monocyte percentage and the occurrence of AECOPD was U-shaped. Patients with monocyte percentage < 7.4% with an absolute count < 0.62 or monocyte percentage > 10% may have a high risk of developing AECOPD.

Previous studies had reported an association between circulating monocytes and FEV1 decline in patients with COPD^19^. Another study had reported that significantly higher monocyte percentage are found in patients with AECOPD than in healthy subjects^15^. These findings indicated that monocytes contributed to the pathogenesis and exacerbation of COPD. According to previous studies, monocyte percentage ranged from 6.2 to 8.1% in patients with COPD and from 6.75 to 7.61% in patients with AECOPD^15,19^. Our findings demonstrated that monocyte percentage range from 5.9 to 11.5% in patients with COPD. Findings from these studies suggested that the normal ranges of monocyte percentage in patients with COPD and AECOPD may be higher than those in healthy subjects (2–8%). Findings on the association between monocyte percentage and AECOPD are unclear. One study reported no significant differences in the monocyte percentage of healthy subjects and patients with COPD at admission or readmission^15^. By contrast, a SHAP summary plot in our previous study reported nonlinear relationships between monocyte percentage and AECOPD^17^. In the present study, we found that the patients with monocyte level < 7.4% or > 10% had a higher risk of AECOPD. Based on these findings, to the best of our knowledge, our study is the first to provide evidence of nonlinear, U-shaped association between monocyte level and the occurrence of AECOPD.

AECOPD are usually associated with viral or bacterial infection and led to acute worsening of symptoms and faster decline in lung function^20^. Monocytes were crucial for controlling and eliminating bacterial and viral infections; however, the recruited monocytes can have deleterious effects and trigger immunopathological reactions under certain conditions^21^. A previous study reported that patients with monocytosis, defined as a blood monocyte level > 10%^22^, experienced respiratory symptoms and suffered from infection more frequently than individuals with normal monocyte percentage^23^. In our study, we found that patients with monocyte percentage of > 10% or < 7.4% had a significantly higher risk of pneumonia than those with medium monocyte percentage. Although no significant differences were noted in respiratory symptoms among the patients in the three groups, the prevalence of dyspnea was higher among those with low and high monocyte percentage. A possible explanation for the difference between the previous study and the present one was that the two populations had distinct endotype differences. We found that, although the low and high monocyte groups both had a high risk of AECOPD, the low monocyte groups had higher neutrophil counts, whereas the high monocyte group had lower neutrophil counts. Based on these findings, we suggested that patients with AECOPD have different endotypes.

Despite the detailed mechanism regarding the association between monocyte level and AECOPD is still unclear, there was evidence to support the positive association between high monocyte level and AECOPD. In previous studies, the monocyte/macrophage levels increased in patients with COPD, these cells do not perform phagocytic and killing functions, resulting in persistent bacterial colonization and necrotic accumulation, which leads to chronic inflammation^24–26^. For example, defective monocyte-derived macrophage phagocytosis was associated with exacerbation frequency in COPD, resulting in pro-inflammatory macrophages that may contribute to disease progression. The possible explanation for the positive association between low monocytes and AECOPD was that there are other cellular mechanisms involved in inflammation in AECOPD patients. Rodríguez‑Guzmán et al. observed an expected increase in the percentage of neutrophils during an exacerbation, which was accompanied by a decrease in the monocyte level^27^. Therefore, neutrophil populations in different patients may be responsible for the nonlinear relationship between monocyte percentage and AECOPD. Neutrophils are the key inflammatory cells present in the bronchial wall and lumen of patients with COPD, recurrent infections and persistent bacterial airway colonization are the drivers of further inflammatory changes^28–31^. In such settings, specific neutrophil degranulation products such as NE have long been implicated for tissue damage repair^29^. These evidence suggested that both monocytes and neutrophils contribute to persistent inflammation in patients with COPD and, sometimes, AECOPD. However, our findings did not directly address the mechanisms underlying the interaction between neutrophils and monocytes in AECOPD. Patients with low and high monocyte percentage may have a higher risk of AECOPD due to respiratory tract infection, which may relate to monocytes or neutrophils, respectively. Thus, owing to the heterogeneity of AECOPD, physicians should comprehensively monitor patients using blood cell parameters instead of relying on a single parameter.

Blood cell parameters were cheap, easy, and quick-to-obtain inflammatory markers and have been widely used in predicting AECOPD. In our studies, we used RCS models to evaluate the nonlinear relationships between AECOPD and the counts of WBCs, including monocytes, eosinophils, neutrophils, and lymphocytes, as well as NLR and ELR. Although previous studies had validated the feasibility of NLR for predicting AECOPD^32^, in the present study, we found nonlinear associations only between the monocyte levels and ELR. This might be attributable to endotypes and the severity of patients with AECOPD. First, prior studies had demonstrated that patients with neutrophilic AECOPD had statistically significant increased NLR values because the attack severity increased but NLR did not correspondingly increase in patients with eosinophilic AECOPD. Thus, NLR was a better predictor in patients with neutrophilic AECOPD^33^. In the present study, we did not distinguish the endotypes of each patient because it was challenging to compare the performance of NLR in predicting AECOPD between two different study populations. Second, previous studies had reported that NLR was a potential predictor of AECOPD events, including invasive mechanical ventilation requirements and mortality^33,34^. However, in our studies, AECOPD was defined as outpatient visits, emergency room visits, or hospital admission at which time systemic steroids were prescribed. Moreover, we only recruited outpatients without a prior history of AECOPD. Furthermore, the model used for evaluating the performance of NLR in predicting AECOPD differs among the studies. As mentioned above, it was challenging to compare the accuracy of NLR in predicting AECOPD between different study populations.

Our study had some limitations. First, the generalizability of the findings must be expanded through further evaluations by using multicenter studies. Second, the impact of comorbidities such as coronary artery disease (which may also lead to high monocyte counts) on the observed association between monocyte percentage and AECOPD was unknown and need to be studied^35^. Third, our study lacked the environmental data of patients; however, the effects of short-term exposure to ambient PM2.5 on the blood cell count have been reported^36^. The effects of air pollution on the observed association between monocyte percentage and AECOPD need further investigation. Fourth, there are different method to evaluate the percentages of WBC populations such as haematology analysers and flow cytometer, which may make percentage vary^37^. Further investigation is required to compare the percentages of monocytes determined by different equipment and their relationship with AECOPD.

## Conclusions

In conclusion, we reported a nonlinear relationship between monocyte level and the occurrence of AECOPD. Patients with monocyte percentage > 10% or < 7.4% with an absolute count < 0.62 were at a high risk of AECOPD. Our findings demonstrated the specific monocyte value for identifying the high risks of AECOPD. This value served as a novel, simple, and inexpensive biomarker in patients with a high risk of AECOPD and should be further investigated in future prospective clinical studies.

## Supplementary Information

Supplementary Information.

## Data Availability

Clinical research data from Changhua Christian Hospital supported the findings of this study. Restrictions apply to the availability of these data and they are therefore not publicly available. These data can only be accessed by Changhua Christian Hospital Big Data center. The datasets used and/or analyzed during the current study are available from the corresponding author on reasonable request.
