# Supplementary material for: Blood monocyte levels predict the risk of acute exacerbations of chronic obstructive pulmonary disease: a retrospective case–control study
Source: Sci Rep. 2022 Dec 6;12:21057. doi: 10.1038/s41598-022-25520-8 (PMC9727121; doi:10.1038/s41598-022-25520-8)

**Supplemental Figure S1. The timeline of the case–control study of 138 cases and 306 controls among COPD patients from JCT DSC-COPD certification program. The AECOPD event was the earliest event from the date of monocyte count collection. The period of study was 1 years after program enrollment.**


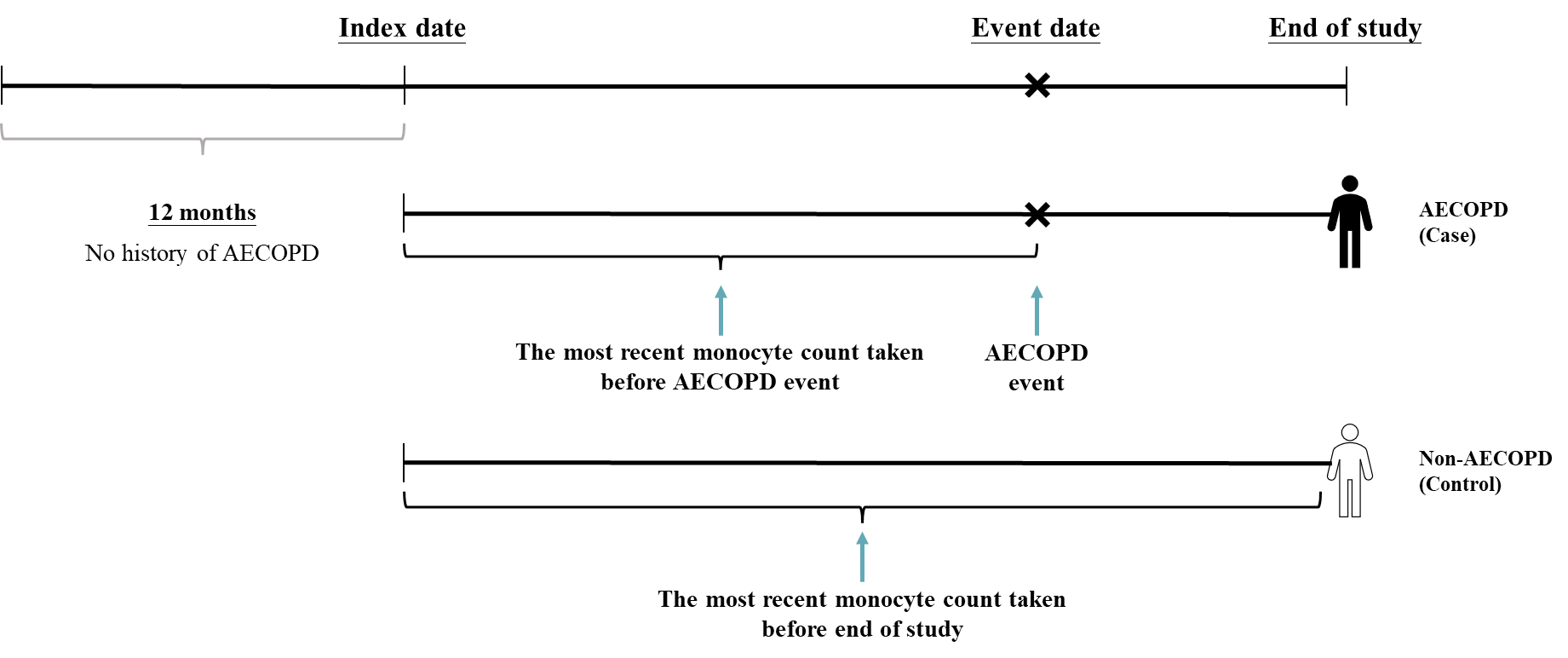


**Supplemental Table S1. The logistic regression for ELS and first-time AECOPD risk**

|  | **Unadjusted logistic model** | | **Adjusted Logistic model** | |
| --- | --- | --- | --- | --- |
|  | **cHR  (95% CI)** | **P-value** | **aHR (95% CI)** | **P-value** |
| Eosinophil to Lymphocyte |  |  |  |  |
| <0.049 | 1.32(0.79,2.2) | 0.288 | 0.71(0.38,1.34) | 0.287 |
| 0.049 to <0.122 | Reference |  | Reference |  |
| >=0.122 | 1.38(0.83,2.3) | 0.212 | 0.92(0.49,1.72) | 0.791 |

The variables in the logistic adjustment model were included as the same as variables of model 1 in Table 2.

**Supplemental Table S2. Adjustment of Odds Ratios for Occurrence of AECOPD Based on Monocyte Percentage Using the Lasso and Ridge Method**

|  | **Lasso logistic model** | | | **Ridge logistic model** | | |
| --- | --- | --- | --- | --- | --- | --- |
|  | **aOR (95% CI)** | **P-value** | **aOR (95% CI)** | | **P-value** |  |
| Monocyte |  |  |  | |  |  |
| Low | 1.29 (0.66,2.54) | 0.453 | 1.33(0.69,2.57) | | 0.393 |  |
| Medium | Reference |  | Reference | |  |  |
| High | 2.78 (1.45,5.34) | 0.002 | 2.28(1.2,4.35) | | 0.012 |  |

Lasso logistic model adjusted for FEV1%, CAT, MMRC, vital signs (DBP, breathing), symptoms (cough, dyspnea, wheezing), medication use (oral LABA, methylxanthines), COPD prescription (COPD dual, COPD triple), comorbidities (HPT, CHF, CPD, sleep disorder, pneumonia, malignancy), and lab data (RBC count, WBC count, Hb); these variables were selected from the lasso regression model with an optimal value of lambda that minimized cross-validation errors. The optimal value of lambda used was 0.014 (Supplemental Figure S2).

Ridge logistic model adjusted for CAT, MMRC, vital signs (breathing rate), symptoms (cough, dyspnea, wheezing), medication use (oral LABA), COPD prescription (COPD dual, COPD triple), comorbidities (HPT, CPD, pneumonia), and lab data (RBC count); these variables were selected using the lasso regression model, with the smallest number of variables that also yields good accuracy. The optimal value of lambda used was 0.07 (Supplemental Figure S2).

All multivariate logistic regression analyses were performed using a backward eliminating process.

**Supplemental Figure S2. The cross-validation error according to the log of lambda**

(A) Lasso regression

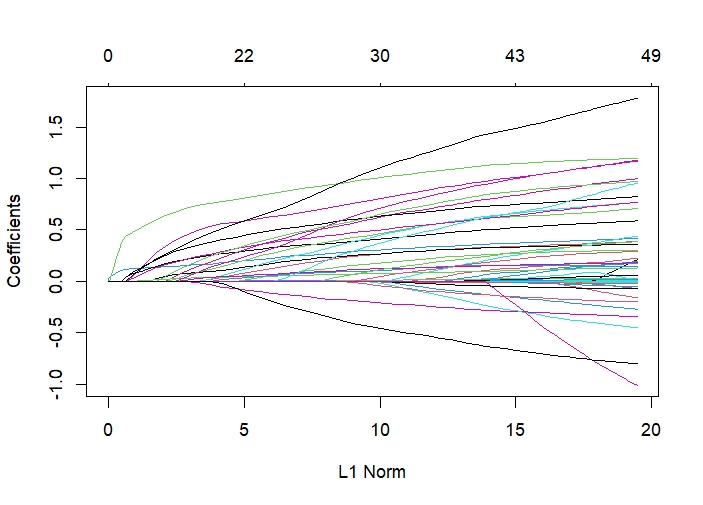

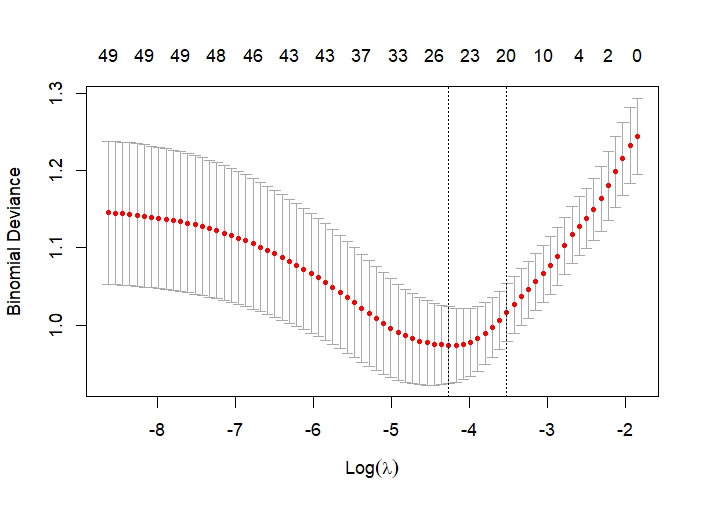


(B) Ridge regression


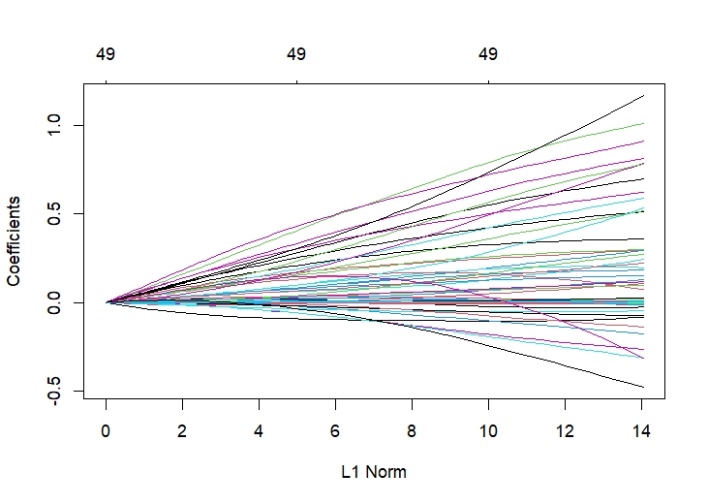

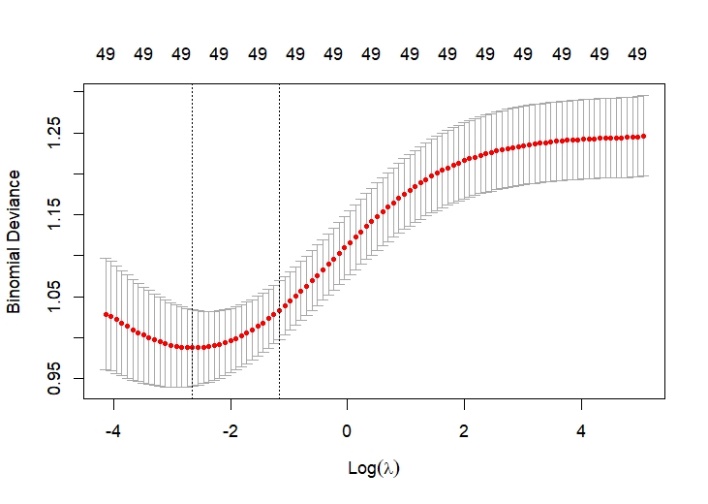

Supplement: Supplementary file 1 — Supplementary Information. [file 41598_2022_25520_MOESM1_ESM.docx]
